# Data on Transfer of Human Coronavirus SARS-CoV-2 from Foods and Packaging Materials to Gloves Indicate That Fomite Transmission Is of Minor Importance

**DOI:** 10.1128/aem.02338-21

**Published:** 2022-03-14

**Authors:** S. Butot, S. Zuber, M. Moser, L. Baert

**Affiliations:** a Société des Produits Nestlé, Nestlé Research, Institute of Food Safety and Analytical Science, Lausanne, Switzerland; Centers for Disease Control and Prevention

**Keywords:** SARS-CoV-2, fomite, food, packaging, transmission

## Abstract

Severe acute respiratory syndrome coronavirus 2 (SARS-CoV-2) infection is mainly transmitted via droplets and aerosols. To evaluate the role of transmission by fomites, SARS-CoV-2-specific data on transfer rates from surfaces to hands and from hands to face are lacking. Here, we generated quantitatively controlled transfer rates for SARS-CoV-2 from food items (lettuce, ham, and vegetarian meat alternative [VMA]) and packaging materials (cardboard and plastic) to gloves using a wet, dry, and frozen viral inoculum and from glove to glove using a wet viral inoculum. For biosafety reasons, the transfer from surfaces to hands and hands to face was simulated by using gloves. The cumulative transfer rate was calculated by using the data from the first transfer experiment, food or packaging material to glove, and combined with the transfer rate obtained from the second transfer experiment from glove to glove. The cumulative transfer rates from lettuce (4.7%) and ham (3.4%) were not significantly different (*P* > 0.05) but were significantly higher (*P* < 0.05) than that from VMA (“wet” or “frozen”). The wet cumulative transfer rate from VMA (1.3%) was significantly higher than the cumulative transfer rate from frozen VMA (0.0011%). No transfer from plastic or cardboard was observed with a dry inoculum. The plastic packaging under wet conditions provided the highest cumulative transfer rate (3.0%), while the cumulative transfer from frozen cardboard was very small (0.035%). Overall, the transfer rates determined in this study suggest a minor role of foods or food packaging materials in infection transmission.

**IMPORTANCE** The observation of SARS-CoV-2 RNA in swab samples from frozen fish packages in China, confirmed only once by cell culture, led to the hypothesis that food contaminated with SARS-CoV-2 virus particles could be the source of an outbreak. Epidemiological evidence for fomites as infection source is scarce, but it is important for the food industry to evaluate this infection path with quantitative microbial risk assessment (QMRA), using measured viral transfer rates from surfaces to hands and face. The present study provides transfer data for SARS-CoV-2 from various types of foods and packaging materials using quantitative methods that take uncertainties related to the virus recovery from the different surfaces into consideration. The transfer data from this model system provide important input parameters for QMRA models to assess the risk of SARS-CoV-2 transmission from contaminated food items.

## INTRODUCTION

Severe acute respiratory syndrome coronavirus 2 (SARS-CoV-2) appeared in Wuhan, Hubei Province, China, in December 2019 (1, 2), from where it spread worldwide, causing the COVID-19 pandemic (3). Transmission occurs by (i) deposition of respiratory droplets and particles on exposed mucous membranes by direct splashes and sprays, by (ii) inhalation of very fine respiratory droplets and aerosol particles, and by (iii) hands that have been directly contaminated with virus-containing respiratory fluids or indirectly via touching virus-contaminated surfaces (4).

The presence of SARS-CoV-2, detected by reverse-transcription PCR (RT-PCR), has been reported on various surfaces (5, 6). However, viral RNA detection by RT-PCR does not necessarily reflect infectivity as detected in cell culture. During the first wave of the COVID-19 pandemic, a study from Israel investigated surface swabs from two hospital isolation units and a quarantine hotel. While viral RNA was detected on 40% to 50% of 97 surface samples from the surroundings of symptomatic COVID-19 patients, none of the surface samples in this study were found to contain an infectious virus in the cell culture assay (7). Similarly, from 26 surface samples from a patient respiratory helmet in an Italian hospital, none induced a cytopathic effect in cell culture (8). One study was able to recover viable SARS-CoV-2 but only from a hospital air sample, not from a surface (9). These results can be explained by the difference in method sensitivity and that not all detectable RNA is infectious, as shown in a study where only SARS-CoV-2 recovered from nonporous surfaces with a threshold cycle (*C_T_*) value of <30 (corresponding to an E gene copy number of ≥10^5^ per mL) yielded SARS-CoV-2 that could be cultured (10).

Few circumstantial data suggest SARS-CoV-2 transmission via fomites. An indirect contact event through a contaminated elevator button was the only epidemiological association found between cluster cases of two families in Guangzhou, China (11). An outbreak at the Qingdao Chest Hospital in China was linked to a hospital suite where transmission may have occurred from a positive patient to a nursing assistant who never met but used the same suite (12). Transmission by fomites was demonstrated in two studies where SARS-CoV-2 infection was observed in hamsters placed in previously contaminated cages (13, 14).

To estimate the SARS-CoV-2 infection risk by fomite transmission, quantitative microbial risk assessment (QMRA) is a useful approach. A key input parameter to estimate this risk is the transfer rate of SARS-CoV-2 experimentally measured by inoculation of the virus onto a surface and transfer to another surface, but these data are lacking. Therefore, transfer data from surrogates such as bacteria, nonenveloped bacteriophages (e.g., MS2, ϕX174, and PRD-1 phage) (15–18), or enveloped phages (e.g., Phi6) (19) have been used. Transfer data for two human coronaviruses, 229E and OC43, are also available (20). The objective of this study was to provide transfer data for SARS-CoV-2 from various food surfaces and packaging materials. We generated transfer data for different real-life scenarios, namely, “wet,” “dry,” and “frozen.” The data indicate that transmission via foods or food packaging materials is of minor importance for SARS-CoV-2 and will help to refine currently available QMRAs.

## RESULTS

### Transfer from food: lettuce, ham, and vegetarian meat alternative.

Table 1 shows the mean SARS-CoV-2 enumeration on gloves after transfer from food and the method recovery values from the glove (recipient) used to calculate the mean percent SARS-CoV-2 transferred from various donor foods to the recipient glove (first transfer), also shown in Table 1. The highest transfer was measured for lettuce at 40.5%, and only 0.0096% transfer was observed for frozen vegetarian meat alternative (VMA) (2 out of 6 replicates were below the limit of quantification [LOQ]). The transfer rates from lettuce and ham (wet) to glove, 40.5% and 28.9%, respectively, were not significantly different (*P* > 0.05) but were significantly higher (*P* < 0.05) than that from VMA (wet or frozen) to glove. The wet transfer rate from VMA to glove (11.3%) was significantly higher (*P* < 0.05) than the transfer rate from frozen VMA to glove (0.0096%).

**TABLE 1:** SARS-CoV-2 enumeration on glove after transfer from food (first transfer), percent recovery from the glove (recipient), and percent transfer of SARS-CoV-2 from food to glove (first transfer)

Matrix^*a*^	Mean ± SD SARS-CoV-2 transferred (log_10_/sample)	Recovery, mean ± SD (%)	Mean % SARS-CoV-2 transferred (CI_low_; CI_high_)
Lettuce wet^a^	5.1 ± 0.1	27.6 ± 13.1	40.5 (14.3; 114.9)
Ham wet^a^	4.9 ± 0.1		28.9 (10.2; 82.2)
VMA wet^b^	4.3 ± 0.1	16.6 ± 5.8	11.3 (4.0; 32.1)
VMA frozen^c^	1.3^*b*^ ± 0.3		9.6 × 10^−3^ (3.4 × 10^−3^; 2.7 × 10^−2^)

a Same letters after names indicate groups of transfer rates that are not significantly different (*P* > 0.05).

b Two out of 6 replicates were <LOQ.

To estimate the risk of SARS-CoV-2 fomite transmission, a second transfer from hands to face needed to be taken into consideration. The second transfer was determined in a separate experiment due to the low virus levels obtained after the first transfer and for which a subsequent transfer would have resulted in virus levels below the LOQ. To do so, the transfer from an inoculated glove to another glove was determined directly after inoculation (wet). The mean percent SARS-CoV-2 transferred from glove to glove was 11.7%, with the following lower (CI_low_) and upper (CI_high_) limits: 4.1 and 33.2 (Table 2). The cumulative transfer rate was then calculated by using the data from the first transfer experiment, food to glove, and combined with the transfer rate obtained from the separate second transfer experiment.

**TABLE 2:** SARS-CoV-2 enumeration on glove after transfer from glove (second transfer), percent recovery from the glove (recipient), and percent transfer of SARS-CoV-2 from glove to glove (second transfer)

Matrix	Mean ± SD SARS-CoV-2 transferred (log_10_/sample)	Recovery, mean ± SD (%)	Mean % SARS-CoV-2 transferred (CI_low_; CI_high_)
Gloves wet	4.5 ± 0.2	27.6 ± 13.1	11.7 (4.1; 33.2)

**FIG 1:**
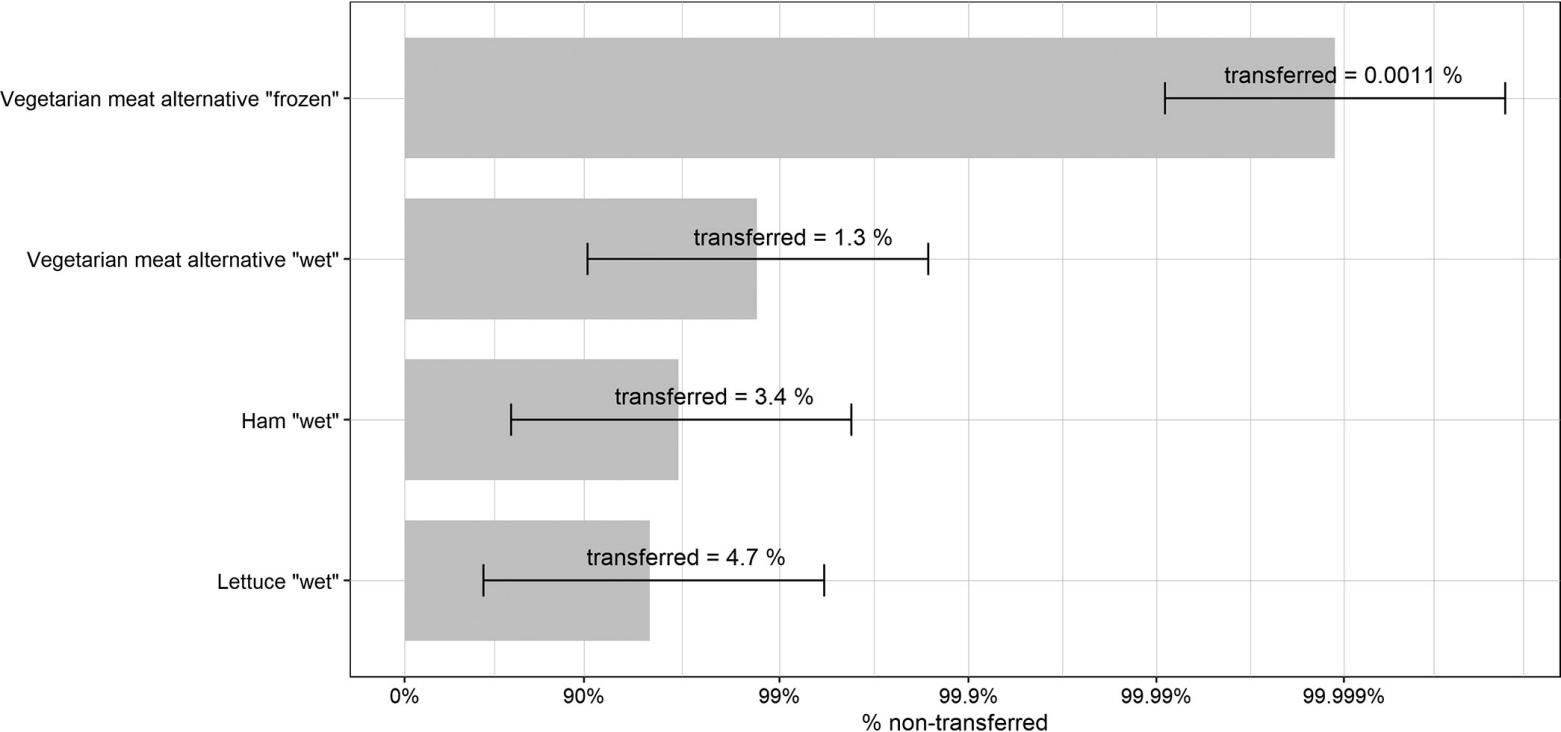
Cumulative transfer rates from foods. The bars represent the percentage of nontransferred SARS-CoV-2 remaining on the donor food on a logarithmic scale, and error bars represent the 95% confidence interval. The percent mentioned on the bars represents the mean percent SARS-CoV-2 transferred with the following lower (CI_low_) and upper (CI_high_) limits: lettuce, 0.6 and 38.1; ham, 0.4 and 27.2; VMA wet, 0.2 and 10.6; and VMA frozen, 1.4 × 10^−4^ and 9.0 × 10^−3^. Each donor surface was inoculated with 6.0 ± 0.3 log_10_ TCID_50_ per 25 cm^2^ of surface.

Figure 1 illustrates the percent nontransferred SARS-CoV-2 particles remaining on the donor foods after the cumulative transfer on a logarithmic scale. The larger the bar, the less transfer took place. On the bars, the average percent SARS-CoV-2 that was transferred from food to the second recipient glove (cumulative transfer) is also indicated. The highest cumulative transfer was measured for lettuce with 4.7%, followed by ham with 3.4%, VMA with 1.3%, and 0.0011% with frozen VMA. The cumulative transfer rates from lettuce and ham were not significantly different (*P* > 0.05) but were significantly higher (*P* < 0.05) than those from VMA (wet or frozen). The wet cumulative transfer rate from VMA (1.3%) was significantly higher than the cumulative transfer rate from frozen VMA (0.0011%).

### Transfer from packaging materials: plastic and cardboard.

Table 3 shows the mean SARS-CoV-2 enumeration on gloves after transfer from packaging material and the method recovery values from the glove (recipient) used to calculate the mean percent of SARS-CoV-2 transferred from various donor packaging materials to the recipient glove (first transfer), also shown in Table 3. Transfer rates were compared directly after viral inoculation (wet), after drying of the inoculum on the packaging material (dry), and after freezing of the inoculum on the packaging material (frozen).

**TABLE 3:** SARS-CoV-2 enumeration on glove after transfer from packaging material (first transfer), percent recovery from the glove (recipient), and percent transfer of SARS-CoV-2 from packaging material to glove (first transfer)^*a*^

Matrix	Mean ± SD SARS-CoV-2 transferred (log_10_/sample)	Recovery, mean ± SD (%)	Mean % SARS-CoV-2 transferred (CI_low_; CI_high_)
Cardboard wet^b^	4.4 ± 0.1	27.6 ± 13.1	9.2 (3.3; 26.3)
Plastic wet^a^	4.9 ± 0.2		25.3 (8.9; 71.8)
Cardboard dry^c^	**1.0**		**0.1** (3.5 × 10^−2^; 0.3)
Plastic dry^c^	**1.0**		**0.1** (3.5 × 10^−2^; 0.3)
Cardboard frozen^c^	3.0 ± 0.2		0.3 (0.1; 0.9)
Plastic frozen^b^	4.5 ± 0.2		10.9 (3.9; 31.0)

a Levels in boldface are below the LOQ. Same letters after names indicate groups of transfer rates which are not significantly different (*P* > 0.05).

The highest transfer was observed for plastic directly after viral inoculation (25.3%) (Table 3). The transfer from cardboard, directly after inoculation (wet) (9.2%), and the transfer from frozen plastic (10.9%) were not significantly different (*P* > 0.05). No transfer was observed from both cardboard and plastic when the viral inoculum was dried before transfer (referred to as dry), since the LOQ was reached. Transfer from frozen cardboard was 0.3% and, thus, not significantly different from plastic and cardboard when the viral inoculum was dried before transfer (*P* > 0.05).

**FIG 2:**
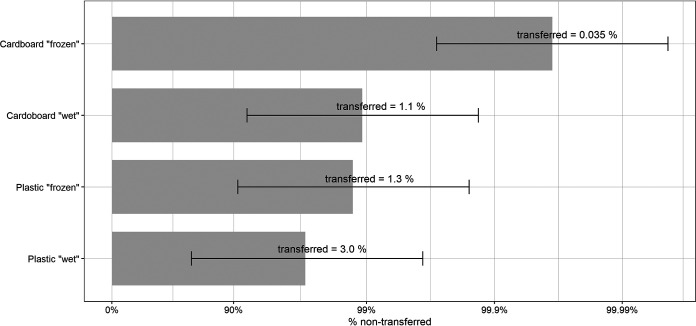
Cumulative transfer rates from packaging materials. The bars represent the percentage of nontransferred SARS-CoV-2 remaining on the donor packaging material on a logarithmic scale, and error bars represent the 95% confidence interval. The percent mentioned on the bars represents the percent SARS-CoV-2 transferred with the following lower (CI_low_) and upper (CI_high_) limits: cardboard wet, 0.1 and 8.7; plastic wet, 0.4 and 23.8; cardboard frozen, 0.4 × 10^−2^ and 0.3; and plastic frozen, 0.2 and 10.3. Each donor surface was inoculated with 6.0 ± 0.3 log_10_ TCID_50_ per 25 cm^2^ of surface.

Using the same working hypothesis as that for food, a second transfer from hands to face needed to be taken into consideration to estimate the risk of SARS-CoV-2 fomite transmission from packaging material. The transfer rate of the wet glove to glove experiment (11.7%) was used, as for the food items (Table 2). Since the LOQ was reached after drying of the viral inoculum onto cardboard or plastic in the first transfer experiment, the cumulative transfer rate using the glove-to-glove transfer was not computed for these samples. For the wet and frozen packaging materials for which a virus transfer to glove was measured in the first transfer, the plastic packaging under wet conditions provided the highest cumulative transfer rate (3.0%), while the cumulative transfer from frozen cardboard was very small (0.035%) (Fig. 2).

### The sum of the percent SARS-CoV-2 transferred and nontransferred.

Controls were included in the transfer experiments to verify the approach and the calculations. For every donor material, the viral concentration after the transfer to the glove was enumerated, taking into consideration the recovery of the donor material, i.e., the percent nontransferred SARS-CoV-2. Additionally, the viral concentration on the recipient material after transfer was enumerated, taking into consideration the recovery of the recipient material, i.e., the percent transferred SARS-CoV-2. In theory, the sum of the percent nontransferred and the percent transferred viral particles should be 100%. Considering the 95% confidence interval, this was the case for all matrices, except for cardboard (wet) (Table 4). This can be explained by the fact that after virus inoculation on the cardboard, the liquid was directly taken up by the cardboard. Consequently, the adsorbed virus was not transferred but could not be efficiently recovered from the donor material during the swabbing.

**TABLE 4:** Percent recovery from the food and packaging material (donor)^*e*^

Matrix	Recovery mean ± SD (%)	Mean ± SD nontransferred (log_10_/sample)	Mean % nontransferred	Mean % transferred^*d*^	Yes^*a*^/no^*b*^ (CI_low_; CI_high_ of the sum)
Lettuce wet	26.5 ± 9.0	5.4 ± 0.1	106.6	40.5	Yes (52; 417)
Ham wet	50.6 ± 24.0	5.3 ± 0.3	34.2	28.9	Yes (22; 179)
VMA wet	6.9 ± 4.2	4.8 ± 0.5	60.4	11.3	Yes (25; 204)
VMA frozen	6.9 ± 4.2	4.8 ± 0.1	93.3	9.6 × 10^−3^	Yes (33; 265)
Carboard wet	16.5 ± 5.5	3.8 ± 0.4	3.5	9.2	No (4; 36)
Plastic wet	28.6 ± 13.2	5.1 ± 0.4	39.1	25.3	Yes (23; 183)
Cardboard dry	0.7 ± 0.4	3.7 ± 0.3	59	0.1^*c*^	Yes (21; 166)
Plastic dry	1.0 ± 0.1	3.9 ± 0.2	73.9	0.1^*c*^	Yes (26; 210)
Cardboard frozen	12.4 ± 13.3	4.8 ± 0.5	84.6	0.3	Yes (30; 241)
Plastic frozen	19.7 ± 14.6	5.2 ± 0.2	71.5	10.9	Yes (29; 234)

a Yes, 100% is within the 95% confidence interval.

b No, 100% is not within the 95% confidence interval.

c Below the limit of quantification.

d Values taken from Tables 1 and 3.

e The SARS-CoV-2 enumeration on the donor after transfer (nontransferred from the donor) was used to calculate the percent nontransferred to the recipient. The sum of the percent nontransferred and the percent transferred was used to verify if the sum of viral particles from donor and recipient is 100%, taking the 95% confidence interval, represented by the CI_low_ and CI_high_, into consideration.

## DISCUSSION

A person touching contaminated food or food packaging and subsequently their face may transfer virus to their nose, mouth, or eyes and get infected without direct contact with an infected person. Persistence of SARS-CoV-2 on various surfaces has been demonstrated (21). SARS-CoV-2 inoculated on processed salmon at 4°C remained infectious for more than 1 week (22). Infectious SARS-CoV-2 was also isolated from swab samples of frozen fish packaging in Qingdao, China (23). Authors of another study speculated that some outbreaks reported in China were initiated by food workers who came into contact with contaminated imported food products when unloading ships, but the alternative infection source from infected crew members of the transporting ships was not explored (24). Based on these observations, Chinese authorities imposed viral RT-PCR-based tests on imported products and their packaging, forcing food manufacturers to put in place additional disinfection steps of their packaging (25).

To evaluate the risk of viral transmission from inanimate surfaces, data on the transfer rates of SARS-CoV-2 from surfaces such as food or food packaging to hands and from hands to face are lacking in the literature. To address this knowledge gap, we conducted laboratory experiments to assess transfer rates of SARS-CoV-2 from foods and their packaging to gloves, used as a substitute for hands (first transfer), and in an independent set of experiments from gloves to gloves, used as a substitute for hands and face (second transfer). Combination of the first and second transfer rates gives a cumulative transfer rate that corresponds to the viral exposure experienced by a susceptible subject. These data can be used as input parameters of QMRAs to estimate infection risks linked with infection transmission from fomites.

Our data show that SARS-CoV-2 transfer under wet conditions (wet) was generally higher than that under dry conditions (dry). Indeed, computation of the second transfer was not needed for the dry cardboard and plastic samples for which the transfer was performed after drying of the viral inoculum, because the LOQ was already reached after the first transfer. This phenomenon was also observed for the transfer of S. aureus from fabrics to finger pads, which was higher between fabrics and moistened finger pads than for dry transfer (15). Transfer efficiencies measured for enteric viruses (murine and human norovirus) dried on stainless discs to gloves were also shown to occur at low efficiencies (0.9 to 3.6%), but the authors did not investigate transfer with a wet inoculum (26).

A recent study showed that transfer of Phi6 (a phage used as a surrogate for enveloped viruses) and MS2 from plastic to finger pads was, on average, 28% and 37%, respectively, with standard deviations of up to 23% (19). For their experiments, the authors dried the viral inoculum on the surface before touching it with the finger pad but with no indication on drying times. In our study, the transfer of SARS-CoV-2 dried for 1 h at room temperature on plastic was below 0.1%. This shows that drying has a big impact on the amount of virus transferred, and small differences in drying times might lead to substantial differences in transfer rates. Another reason for the discrepancy with the study from Anderson and Boehm (19) might be linked to the different surface materials, such as plastic types, leading to stronger adhesion and, thus, less transfer of some viruses. A correlation between stronger adhesion of MS2 on polyvinyl chloride (PCV) leading to poorer recoveries was shown previously (27). In this context it is also important to point out that in our study, cardboard provided the lowest transfer efficiencies (maximum mean transfer of 9.2%) under wet conditions. This low rate is explained by the porosity of the surface, as also described for the transfer of bacteria or bacteriophages (16). To address this potential issue, the data analysis in our study was done to calculate the mean transfer including uncertainties related to the virus recovery from the donor and recipient material.

Another difference between our study and other reported studies is the use of gloves compared to finger pads (17–19). The fact that we had to use gloves for biosafety reasons represents a limitation of this study. Future work should include comparison of transfer with potential surrogates such as human coronavirus HCoV-229E or bacteriophage Phi6, which subsequently could be used for transfer experiments with hands and finger pads of human volunteers (19, 28). This would also allow for a deeper assessment of the impact of organic material (e.g., proteins and fat from food surfaces or bodily fluids) and salt concentrations on the transfer rates of the different viruses, as a recent study showed that the human coronavirus OC43 was transferred more efficiently from contaminated hands to contact surfaces in the presence of fecal material (20).

QMRAs estimating the risk of SARS-CoV-2 transmission by fomites are available (29–32). In these QMRAs, the parameter representing the viral transfer from surface to hands was taken from bacterial and phage transfer data (15, 16, 33, 34). Harvey and coworkers (29) estimated the risk of SARS-CoV-2 transmission from high-touch surfaces using transfer efficiencies derived from MS2 transfer data at a high relative humidity (40 to 65%). The transfer data from steel and acrylic to hands were 37.4% and 79.5%, on average (16). Using these input parameters, the risk of infection from touching a contaminated surface in a community setting was estimated to be less than 5 in 10,000 (29) and less than 1.6 in 10,000 or lower, depending on the virus prevalence rate (30). Pitol et al. (30) indicated that the model parameters mostly influencing the estimated infection risk are the transfer efficiency between the surface and the hands and the concentration of SARS-CoV-2 in saliva and respiratory droplets. By using our data for SARS-CoV-2, the assessment of the risk of infection would be more realistic and even lower. Another QMRA estimated the SARS-CoV-2 infection risk of a susceptible worker in a frozen food packaging facility from contact with contaminated packaging to be 1.5 × 10^−3^ per 1-h period when no specific standard food industry infection control measures were put in place (32). Such measures, namely, handwashing and masks, substantially reduced the risk by 99.4%. These examples show how important QMRA frameworks are to estimate the SARS-CoV-2 risk of infection by fomite transmission and to evaluate the efficiency of mitigation strategies.

The data generated in the present study represent key input parameters, since experiments were carried out with SARS-CoV-2 and the cumulative transfer (from food/packaging materials to glove and from glove to glove) was included in the experimental setup. The highest cumulative transfer rate of 4.7% was measured for lettuce. No transfer from dry plastic and cardboard (dry inoculum) was observed. In the absence of a dose-response model, it is currently not possible to determine with certainty if these low transfer rates lead to virus loads that are below the infectious dose. However, in most cases viral inoculum will have time to dry, and our results show that the risk with dry inoculum becomes negligible. The outcome of our study is in agreement with the study conclusions of Sobolik and coworkers (32), indicating that the risk linked to cold-chain food and food packaging contamination as the possible origin of COVID-19 resurgence in China (24, 25) is very low and, hence, surface disinfection of food packaging would not be useful to control SARS-CoV-2 transmission.

## MATERIALS AND METHODS

### Virus and preparation of suspension.

SARS-CoV-2, kindly provided by Isabella Eckerle (Geneva University Hospitals, Center for Emerging Viral Diseases), was propagated, assayed, and titrated on kidney African Green Monkey Vero C1008 (Vero 76, clone E6, and Vero E6) (ECACC 85020206) cells as described previously (28). Viral stock was purified and concentrated by a polyethylene glycol precipitation (0.25 volume of 5× polyethylene glycol-NaCl solution) as described in ISO-15216 (35). The pellets were resuspended in phosphate-buffered saline (PBS) (D8662; Sigma) with salts (MgCl_2_ and CaCl_2_) in the attempt to mimic as closely as possible the salt content of human bodily fluids. Viral titer, determined as the 50% tissue culture infective dose (TCID_50_) per milliliter, as described previously (36), was 7.0 ± 0.3 log_10_ TCID_50_/mL for SARS-CoV-2.

### Samples.

Butterhead lettuce, cooked ham, and chilled VMA, round portions (similar to meat balls but based on soy and wheat proteins), were purchased at a local distributor in Lausanne. The plastic foil samples were taken from the top seal of the tray containing the VMA portions. The cardboard samples were from the outer package of the VMA portions. Nitrile gloves (97613; Kimtech) were used to simulate the human hands and face in the transfer experiments. For all matrices, 25-cm^2^ (5 cm by 5 cm) samples were prepared and fixed onto petri dish lids, except for the VMA, where portions of 15 g were put into a petri dish.

### Study design.

Our study was designed to mimic virus transmission from fomites to a person touching a contaminated food or packaging surface and subsequently touching the face. To simulate this virus transmission, two transfer experiments were carried out. The first transfer experiment was done where the inoculated foods or packaging materials, i.e., the virus donor surfaces, were put in contact with a glove, i.e., the recipient surface (hand). Gloves were used instead of hands, since it was not possible to use hands of volunteers for biosafety reasons. The second transfer experiment simulated the transfer from hand to face. To simulate the second transfer, an inoculated glove was put in contact with a glove, i.e., the donor surface. The cumulative transfer rates were calculated from the first and second transfer experiments. The transfer rates from food or packaging materials were determined by the calculation of transfer rates obtained from two separate transfer experiments, because the aim was to obtain quantitative data. This was only achievable with two single transfers due to the low transfer rates in the first experiment, meaning that virus levels would have been below the LOQ if the second transfer had been done directly after the first transfer.

Different transfer conditions were evaluated in our study. Wet transfer corresponds to a transfer executed directly after viral inoculation at room temperature on the donor surface, meaning that the inoculum was still wet. Frozen transfer corresponds to a transfer performed with the donor surface inoculated at room temperature and immediately after inoculation frozen for 24 h at −20°C. A dry transfer is a transfer carried out with the inoculated donor stored 1 h at room temperature, meaning that the inoculum was dry.

**FIG 3:**
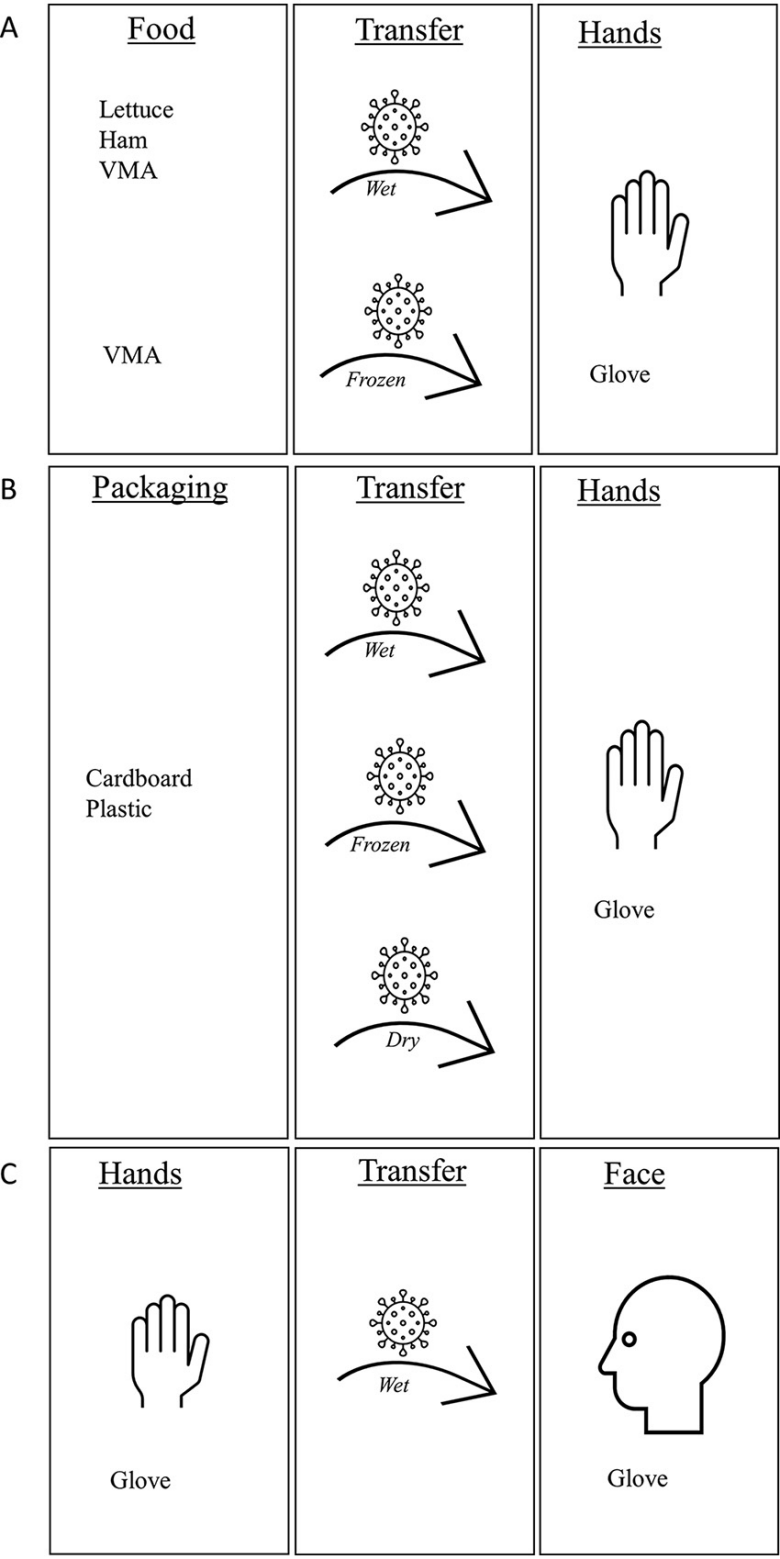
Study design of the cumulative transfer from food to glove (first transfer) (A), from packaging materials to glove (first transfer) (B), and from glove to glove (second transfer) (C). The cumulative transfer rate was calculated by using the data from the first transfer experiment (A or B) and combined with the transfer rate obtained from the separate second transfer experiment (C).

For the food-to-glove transfers, the wet condition was applied for lettuce, ham, and VMA, and the frozen condition was applied for frozen VMA (Fig. 3A). The second transfer from glove to glove was realized using the wet condition (Fig. 3C).

For the first transfer from packaging materials to glove (Fig. 3B), all the transfer conditions (wet, frozen, and dry) were evaluated. For the cumulative transfer, the wet transfer rate from glove to glove (Fig. 3C) was combined with the ones obtained from packaging to glove under the wet and frozen conditions. For the cardboard and plastic samples for which the transfer was performed after drying of the viral inoculum (dry) and for which no transfer was observed, since the LOQ was reached after the first transfer, a second glove-to-glove transfer was not computed.

To verify the approach and the calculations of our study design, controls were included in the transfer experiments. The virus concentration after transfer on the donor (percent nontransferred) and on the recipient (percent transferred) was enumerated. It was verified if the sum of percent nontransferred and percent transferred corresponded to 100%. In practice, the results are considered equal to 100% if the 100% is within the 95% confidence interval, taking the variability of the replicates and the variability of the recovery rate into consideration.

### Inoculation with SARS-CoV-2.

One hundred microliters of SARS-CoV-2 (6.0 ± 0.3 log_10_ TCID_50_), which corresponds to viral loads in saliva of infected patients (37, 38), was spotted (droplets of 5.2 ± 0.4 μL) directly on the sample surface using a technique described previously (39). Spot inoculation was selected, as this method showed more consistent results and mimics natural spots of contamination (39). For the frozen condition, the samples were inoculated and immediately placed at −20°C for 24 h prior to the transfer experiments. For the dry transfer condition, the inoculum was dried for 1 h in a biosafety cabinet at room temperature prior to the transfer. Based on visual inspection, 1 h was the minimum time required to have a dry inoculum on the plastic surfaces.

### Transfer from donor to recipient matrices.

The transfer from lettuce, ham, cardboard, plastic, or glove matrices to glove was done by putting in contact the donor and the recipient surfaces followed by a finger pressure for 10 s (40). For the VMA to glove, the approach followed was the same, except that a 4-kg weight was applied for 10 s instead of finger pressure.

### Virus recovery from samples.

Viruses were recovered from 25 cm^2^ of lettuce, ham, plastic, cardboard, or glove by intensively swabbing the surface using a cotton-tipped swab (115-1881; VWR) predipped in infection medium consisting of Eagle’s minimum essential medium (EMEM) (30-2003; ATCC) supplemented with 2% fetal bovine serum (FBS) (30-2020; ATCC) and 1% penicillin-streptomycin (100×) (P0781; Sigma). The swab was transferred to a 1.5-mL tube containing 0.5 mL of infection medium. The plastic part of the swab was cut to close the tube, and the tube was vortexed vigorously for 1 min to release the viruses. For VMA, viruses were recovered from samples using the ISO 15216 virus extraction method (leaf, stem, and bulb vegetables protocol), with slight modifications. The pH of the Tris-glycine-beef extract (TGBE) buffer was 7.0 instead of 9.5, as optimized during the method evaluation, and no process control was added. The chloroform-butanol clarification step was not performed. Before enumeration, concentrated samples were decontaminated by sequential filtering through 0.45-μm and then 0.22-μm spin centrifuge tube filters (Corning, New York) pretreated with 300 μL of phosphate-buffered saline (pH 7.2 ± 0.2) containing 10% fetal calf serum (41). The recovered viruses from all matrices were 5-fold serially diluted and enumerated by determining the TCID_50_ (36). Six replicates from two different days were obtained for each transfer experiment.

### Method recovery.

For each matrix and inoculation condition (wet, dry, and frozen), the method recovery rates were determined by analyzing 6 replicates. For the first transfer from VMA to glove, the recovery rate from the glove, which acted as a recipient, was determined, taking into consideration the residues from the VMA deposited on the glove surface. For this, prior to the viral inoculation, the glove was put in contact with the VMA with a 4-kg weight for 10 s.

### Data analysis. (i) Transfer rate from food or packaging material to glove (first transfer).

The transfer rate for each matrix/transfer combination was calculated as:

(1) $$\text{Transfer rate=}\frac{{\text{measured viral concentration}}_{\text{after transfer on recipient}}{\text{/method recovery rate}}_{\text{on recipient}}}{\text{virus inoculum}}$$

In equation 1, the virus inoculum was 6.0 ± 0.3 log_10_ TCID_50_, and the method recovery rate on the recipient (glove without or with VMA residues) was calculated as:

(2) $${\text{Method recovery rate}}_{\text{on recipient}}\text{ = }\frac{{\text{measured viral concentration}}_{\text{on recipient}}}{\text{virus inoculum}}$$

In equation 2, the virus inoculum was 6.0 ± 0.3 log_10_ TCID_50_, and putting equation 2 into equation 1, the transfer rate equation becomes:

$$\text{Transfer rate=}\frac{{\text{measured viral concentration}}_{\text{after transfer on recipient}}\text{/}\frac{{\text{measured viral concentration}}_{\text{on recipient}}}{\text{virus inoculum}}}{\text{virus inoculum}}\text{=}\frac{{\text{measured viral concentration}}_{\text{after transfer on recipient}}}{{\text{measured viral concentration}}_{\text{on recipient}}}$$

As the measured TCID_50_ viral concentrations are not normally distributed, the computations of the transfer rates were performed using the log_10_ unit and are therefore expressed as log_10_ reductions:

Log10(measured viral concentrationafter transfer on recipientmeasured viral concentrationon recipient)=log10(measured viral concentrationafter transfer on recipient)- log10(measured viral concentrationon recipient)

For each matrix-transfer combination, the mean log_10_ reduction on the recipient was calculated from the 6 replicates.

To express the transfer rate as a percent, the following calculation was performed:

$${\text{\% transferred = 10}}^{\text{log mean reduction}}$$

To determine the percent nontransferred on the donor, the following equation was applied:

$$\text{\% nontransferred=}\frac{{\text{measured viral concentration}}_{\text{after transfer on donor}}}{{\text{measured viral concentration}}_{\text{before transfer on donor}}}\times \text{ 100}$$

### (ii) Ninety-five percent confidence interval.

The 95% confidence interval was determined by mean (log_10_ reduction) ±*qt*(0.975, *df* = 69) ×SD(mean log_10_ reduction). In this equation, *qt*(0.975,*df*=69) corresponds to the 97.5% quantile of a Student *t* law with 69 degrees of freedom and SD(mean log_10_ reduction) to the square root of SD(transfer)^2^ + SD(recovery)^2^. SD(transfer) is the pooled standard deviation of the transfer experiments. SD(recovery) is the pooled standard deviation of the recovery experiments.

### (iii) Sum of the percentages of transferred and nontransferred virus.

To verify our experimental approach, we calculated the sum of the percent nontransferred virus particles left on the donor and the percent transferred virus particles deposited on the recipient. The sum should be 100%, taking the 95% confidence interval into consideration, which was calculated for each matrix-transfer combination. The confidence intervals of each sum were calculated by combining the variances of the percent transferred viral particles measured on the recipient and the percent nontransferred viral particles measured on the donor after transfer.

### (iv) Statistical significance.

The determination of the statistical significance was based on *t* test using the appropriate variance from measurements done on recipients after transfer, i.e., SD(transfer)^2^. When comparing transfer data from VMA with transfer data from other food matrices, the appropriate variance was SD(transfer)^2^ + SD(recovery)^2^, as the recipient matrices were different (glove with residues from the VMA).

### (v) Cumulative transfer rates from food or packaging materials to glove and from glove to glove.

The cumulative transfer rate from food or packaging material to glove (first transfer) and that from glove to glove (second transfer) was calculated by the sum of the mean (log_10_ reduction) obtained in the first transfer and the second transfer, which is equivalent to the multiplication of the two transfer percentages. On the log scale, the means of all transfer rates are normally distributed; therefore, the sum of them are also normally distributed with a mean equal to the sum of the means and a variance equal of the sum of variances. These values were used to compute the associated confidence interval.

All statistical analyses were performed with the software R version 4.0.2 (202-06-22) (42).
